# Spatially Organized Human Ovarian Spheroids Instruct Endometrial Morphogenesis

**DOI:** 10.1002/advs.76538

**Published:** 2026-07-09

**Authors:** Maria João Sousa, Silke De Vriendt, Lixian Liu, Thalles Fernando Rocha Ruiz, Hugo Vankelecom, Christiani Andrade Amorim

**Affiliations:** ^1^ Pôle de Recherche en Physiopathologie de la Reproduction Institut de Recherche Expérimentale et Clinique (IREC) Université Catholique de Louvain Brussels Belgium; ^2^ Laboratory of Tissue Plasticity in Health and Disease Cluster of Stem Cell and Developmental Biology Department of Development and Regeneration KU Leuven Leuven Belgium

**Keywords:** endometrium organoids, follicle‐like spheroids, Ovarian‐endometrial axis, reproductive bioengineering, steroidogenesis

## Abstract

The human ovarian‐endometrial axis operates through coordinated, dynamic endocrine signaling that cannot be faithfully reproduced by static hormone supplementation. While endometrial organoids (EMOs) respond to exogenous estradiol and progesterone, whether a spatially organized human ovarian endocrine unit can instruct epithelial morphogenesis through physiologically integrated signaling remains unknown. Here, we engineered a multilayered human follicle‐like spheroid composed of primary granulosa cells and stromal‐derived theca‐like cells (SPHEGaT), recreating architectural and steroidogenic features of the ovarian follicle. These constructs generated sustained biologically active concentrations of estradiol and progesterone while maintaining high viability and low hypoxic burden. When co‐cultured with EMOs, SPHEGaTs induced progressive epithelial remodeling characterized by folding morphogenesis, increased progesterone receptor expression, and upregulation of secretory/progesterone‐responsive genes including PAEP, SPP1 and HSD17B2. Strikingly, partial steroid depletion did not abolish organoid folding, whereas static supplementation with exogenous estradiol and progesterone failed to restore the differentiation phenotype. These findings suggest that endometrial remodeling is influenced not merely by hormone concentration, but also by additional signaling cues present within the SPHEGaT‐derived microenvironment. Together, this work establishes a human 3D platform in which integrated ovarian‐derived signaling supports epithelial morphogenesis, providing new framework for studying ovarian‐endometrial communication and highlighting the limitations of hormone‐only models in reproductive bioengineering.

## Graphical abstract

A spatially organized human follicle‐like spheroid (SPHEGaT) composed of granulosa cells (GCs) and stromal‐derived theca‐like cells (TLCs) establishes sustained steroidogenesis in vitro. When co‐cultured with human endometrial organoids (EMOs), estradiol (E2) and progesterone (P4) exchange induces secretory/progesterone‐responsive differentiation, increased PGR, PAEP, SPP1 and HSD17B2 expression.

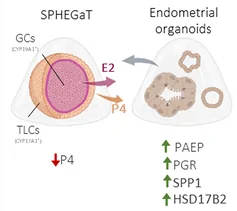

## 1 Introduction

The human ovarian‐endometrial axis operates through highly coordinated endocrine signaling that is intrinsically dynamic, spatially organized, and context‐dependent. Across the menstrual cycle, temporally regulated fluctuations in estradiol (E2) and progesterone (P4) orchestrate epithelial proliferation, differentiation, and the acquisition of uterine receptivity. Disruption of this finely tuned endocrine interplay contributes to infertility, implantation failure, and hormone‐dependent endometrial pathologies [1, 2, 3, 4, 5]. Despite its central physiological importance, the human ovarian‐endometrial interface remains experimentally inaccessible in a controlled and fully human setting.

In vivo, ovarian steroidogenesis is not merely a biochemical process but a spatially organized cellular program. Theca cells (TCs) and granulosa cells (GCs) interact within the three‐dimensional (3D) architecture of the follicle to generate androgens, P4 and E2 [6]. These hormones are delivered to the endometrium in a temporally dynamic manner, where epithelial and stromal compartments integrate steroid gradients into coordinated proliferative and secretory responses. While E2 drives epithelial expansion, P4 reprograms the tissue toward secretory differentiation, decidual competence, and receptivity‐associated gene networks [7]. Importantly, this process depends not only on hormone concentration, but on the integrated microenvironment in which endocrine and paracrine signals are produced.

Three‐dimensional endometrial organoids (EMOs) have substantially advanced the modeling of uterine epithelial biology. EMOs recapitulate glandular architecture, maintain hormone responsiveness, and can undergo secretory‐like differentiation upon exposure to E2 and P4, including induction of canonical markers such as P4‐associated endometrial protein (PAEP) and secreted phosphoprotein 1 (SPP1) [8, 9, 10, 11]. Yet, these systems largely rely on static exogenous hormone supplementation, which does not replicate the dynamic and tissue‐integrated nature of ovarian endocrine signaling observed in vivo.

Parallel advances in ovarian bioengineering have enabled reconstruction of steroidogenic units using spheroids and alginate‐based microcapsules, underscoring the importance of extracellular matrix (ECM) cues and three‐dimensional organization for physiological hormone production [12, 13, 14, 15]. However, these engineered ovarian constructs rely on non‐human cells, and existing attempts to model ovarian‐uterine crosstalk either employ animal follicles, simplified human monolayer cultures, or multichambered microfluidic systems lacking physiologically organized human ovarian architecture [16, 17, 18]. Approaches using follicular fluid or conditioned media provide indirect endocrine input but do not offer controlled, sustained steroid production from a defined human ovarian unit [19]. Consequently, a critical gap persists: the absence of a spatially organized, fully human ovarian endocrine construct capable of instructing endometrial morphogenesis through integrated signaling.

Here, we hypothesized that recreating the 3D architecture of the human follicle would generate an endocrine microenvironment capable of driving endometrial differentiation beyond what can be achieved by static hormone supplementation. To test this, we engineered a multilayered human spheroid composed of primary GCs and stromal‐derived theca‐like cells (SPHEGaT), recapitulating key features of follicular organization and steroidogenic competence. We then established a human 3D co‐culture system combining SPHEGaTs with EMOs to model secretory‐phase ovarian‐endometrial communication in vitro. This platform enables ovarian‐derived signaling to direct epithelial remodeling and provides a tractable framework to investigate ovarian‐endometrial communication in reproductive biology and hormone‐responsive disorders.

## 2 Results

### 2.1 Spatially Organized Assembly of GC and TLCs Generates a Follicle‐Mimetic Endocrine Unit

To reconstruct a human ovarian endocrine microenvironment, we first established a spatially organized spheroid combining primary GCs and stromal‐derived theca‐like cells (TLCs). Immunofluorescence analyses confirmed the steroidogenic identity of each cellular compartment prior to assembly. GCs isolated from IVF patients expressed CYP19A1 (aromatase) and the follicle‐stimulating hormone receptor (FSHR), whereas TLCs differentiated from ovarian stromal cells expressed CYP17A1 and CD13 (Figure 1A), consistent with their respective roles in androgen synthesis and aromatization.

**FIGURE 1:**
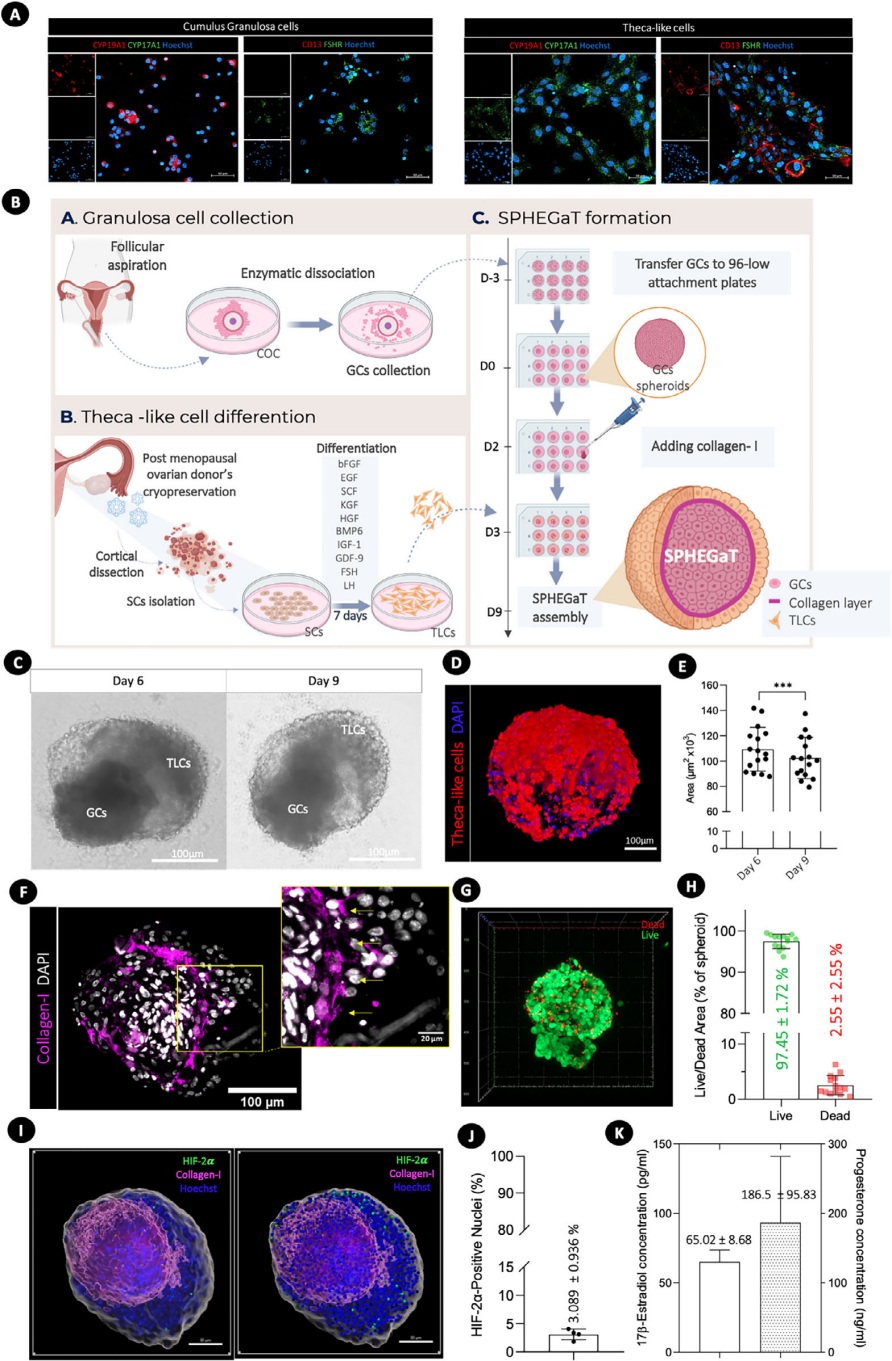
Development and a characterization of theca‐granulosa spheroids. (A) Immunofluorescence analysis of TLCs and GCs. Representative images of CYP19A1 (left, red), CD13 (right, red), CYP17A1 (left, green) and FSHR (right, green). Nuclei are counterstained with Hoechst (blue). Scale bar: 50 µm. (B) Schematic representation of cell preparation for spheroid construction. A‐ GCs were isolated from IVF‐derived COCs by enzymatic dissociation. SCs were isolated from postmenopausal ovarian cortex and differentiated into TLCs. GCs were aggregated in low‐attachment plates, embedded in collagen I, and combined with TLCs to generate the SPHEGaT. COC, cumulus‐oocyte complex; GCs, cumulus/granulosa cells; SCs, stromal cells; TLCs, theca‐like cells; SPHEGaT, spheroids of endocrine granulosa and theca cells. (C) Brightfield images of spheroids at day 6 and day 9 (end of culture), showing granulosa and TLC organization. Scale bar: 100 µm. (D) Fluorescence images showing TLCs labelled with a cell tracker (red) and nuclei counterstained with DAPI (blue). Scale bar:100 µm. (E) Quantification of spheroid area (×10^3^ µm^2^) from day 6 to day 9. Data represent *n*  = 3 independent biological replicates; all technical replicates are included. Bars represent mean ± S.D. ^***^ *P* < 0.001. (F) Immunofluorescence analysis of spheroids showing layered organization. Collagen I is shown in magenta and nuclei are counterstained with DAPI (white). Scale bar:100 µm. (G) Three‐dimensional live/dead representation of spheroids. Live cells are shown in green and dead cells in red. (H) Quantification of live and dead areas expressed as a percentage of the total spheroid area. Bars represent mean ± S.D. (I) Three‐dimensional representation of spheroids showing collagen I (magenta), nuclear HIF‐2α (green) and nuclei counterstained with Hoechst (blue). Scale bar: 80 µm.  (J) Quantification of HIF‐2α‐positive nuclei expressed as a percentage (%) of total cells. (K) Quantification of 17β‐estradiol and progesterone in culture media at day 9. Estradiol is expressed in pg ml^−1^ (left) and progesterone in ng ml^−^^1^ (right). Data represent *n*  = 3 independent biological replicates and are shown as mean ± S.D.

Under low‐adhesion conditions, GCs spontaneously self‐organized into compact 3D aggregates within 3 days, forming a defined core structure. Subsequent addition of TLCs resulted in progressive peripheral organization around the GC core, generating a multilayered architecture reminiscent of the native follicular arrangement (Figure 1B–D). Quantitative analysis demonstrated a significant reduction in spheroid area over time (Figure 1E), reflecting coordinated tissue compaction and structural stabilization rather than uncontrolled aggregation.

Because basement membrane integrity is critical for follicular organization and steroidogenic coordination, we introduced a thin collagen type I interlayer between the GC core and surrounding TLCs. This coating reproducibly enhanced structural stability and better supported steroidogenic activity relative to spheroids cultured without the collagen layer (Figure S1). The collagen interface promoted a more coherent multilayered configuration (Figure 1F), enabling the establishment of a spatially defined endocrine unit that preserves compartmental organization.

Together, these results demonstrate that primary human GCs and stromal‐derived TLCs can be assembled into a stable, multilayered spheroid that recapitulates key architectural features of the ovarian follicle, providing a structurally organized foundation for integrated steroidogenic function.

### 2.2 Architectural Stability Supports Sustained Endocrine Competence

For a spatially organized endocrine unit to exert instructive effects, structural integrity and metabolic stability are essential. Following TLCs incorporation, SPHEGaTs were maintained in culture for another 6 days to allow full compaction and architectural maturation. At the end of this period, constructs exhibited a predominance of viable cells without detectable focal necrosis (Figure 1G). Quantitative analysis confirmed that 97.45 ± 1.72% of the spheroid area was viable, with only 2.55 ± 2.55% classified as non‐viable (Figure 1H), indicating robust cellular maintenance within the compact 3D configuration.

Because oxygen diffusion can limit function in dense aggregates, we evaluated hypoxic stress using HIF‐2α immunostaining. Three‐dimensional reconstruction demonstrated the absence of a hypoxic core (Figure 1I), and only 3.09 ± 0.94% of nuclei were HIF‐2α‐positive (Figure 1J), indicating that spatial organization did not compromise oxygenation. Thus, the multilayered configuration sustains structural compaction without inducing metabolic stress.

We assessed whether architectural integrity translated into functional endocrine output. Media collected from SPHEGaTs (pooled from eight spheroids per replicate) contained endogenous concentrations of E2 (65.02 ± 8.68 pg mL^−1^) and P4 (186.5 ± 95.83 ng mL^−1^) (Figure 1K). Together, these findings demonstrate that spatial assembly of human GCs and TLCs generates a viable, oxygenated, and steroidogenically competent endocrine unit.

### 2.3 SPHEGaT‐Derived Endocrine Signaling Drives Epithelial Morphogenesis Without Altering Proliferative Capacity

We next investigated whether this spatially organized ovarian unit could instruct endometrial architecture. Brightfield imaging revealed marked structural divergence between EMO monocultures and EMOs exposed to SPHEGaTs. While monocultured organoids retained predominantly spherical, cyst‐like morphologies, co‐cultured EMOs progressively developed folded, lobulated, and optically denser structures over 6 days (Figure 2A), indicative of epithelial remodeling.

**FIGURE 2:**
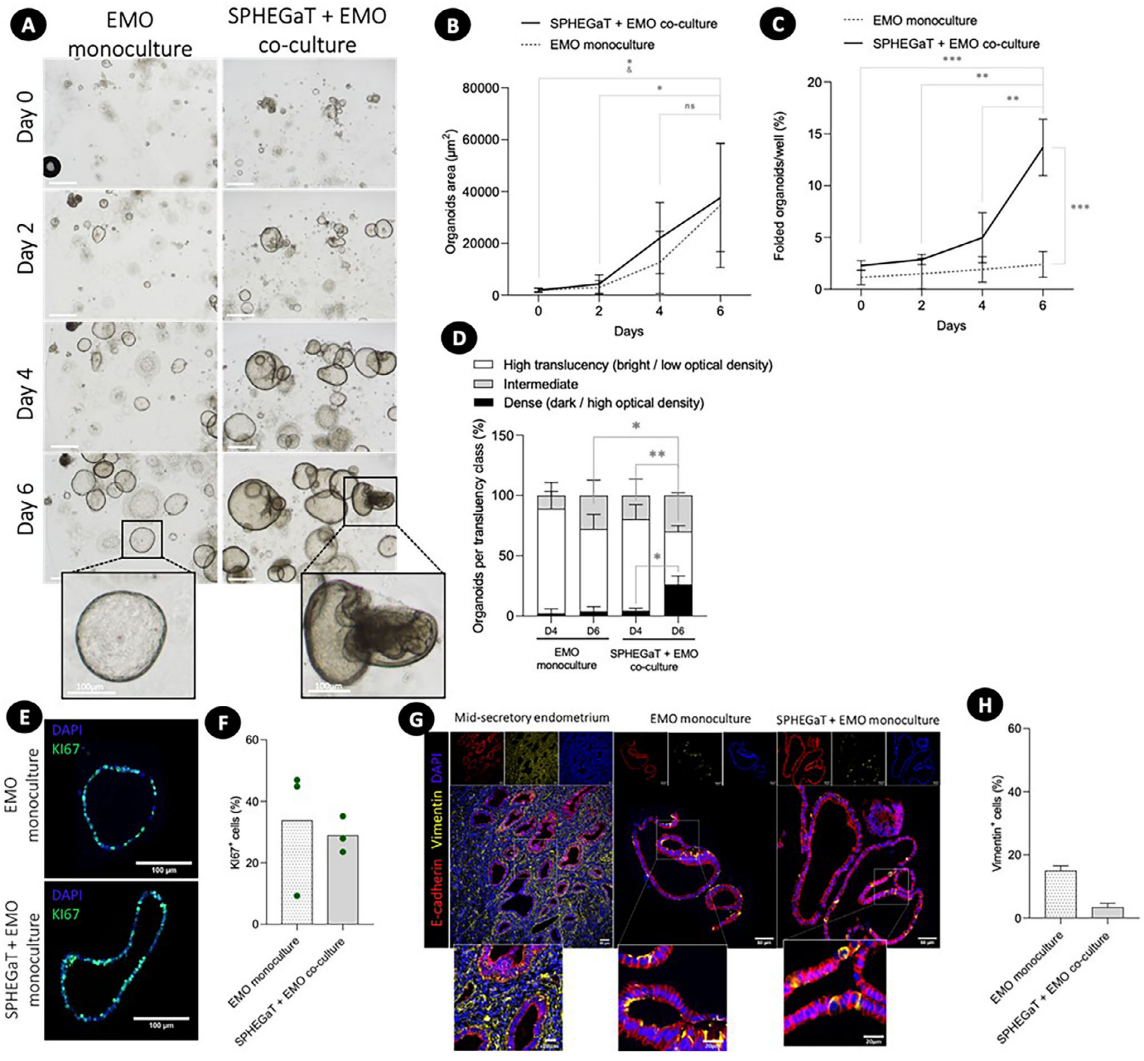
Growth, morphology and epithelial organization of EMOs in monoculture and co‐culture conditions. (A) Brightfield images showing the formation and growth of EMOs over time in monoculture (left panel) and in co‐culture (right panel) over 6 days in culture. Scale bars: 200 µm. Insets show higher‐magnification views of representative organoids. Scale bars: 100 µm. (B)  Quantification of EMOs’ area. Statistical analysis was performed using two‐way ANOVA followed by multiple comparisons testing. ^*^ *P* < 0.05 for SPHEGaT‐EMO co‐culture, & *P* < 0.05 for EMO monoculture, and ns for non‐significant.  *n* = 3 biological replicates. (C) Quantification of organoid folding. Statistical analysis was performed using two‐way ANOVA followed by multiple comparisons testing. ^**^ *P* < 0.01 and ^***^ *P* < 0.001. *n* = 3 biological replicates. (D) Quantification of organoid circularity over the culture period. Lower circularity values indicate increased organoid folding. Statistical analysis was performed using two‐way ANOVA followed by multiple comparisons testing. ^*^ *P* < 0.05 for the SPHEGaT‐EMO co‐culture condition over time, and ^**^ *P* < 0.01 for comparisons between EMO monoculture and SPHEGaT‐EMO co‐culture. n = 3 independent biological replicates. (E)  Quantification of EMO translucency over the culture period. Statistical analysis was performed using two‐way ANOVA followed by multiple comparisons testing. ^*^ *P* < 0.05 and ^**^ *P* < 0.01. *n* = 3 independent biological replicates. (F) Immunofluorescence analysis of EMOs showing proliferative cells stained for KI67 (green) and nuclei counterstained with DAPI (blue). Representative sections are shown. Scale bar: 100 µm. (G) Quantification of KI67‐positive cells expressed as a percentage of total nuclei. Each dot represents the average per replicate. Bars represent mean ± S.D. *n* = 3 biological replicates. (H) Immunofluorescence analysis of epithelial organization showing E‐cadherin (red), vimentin (green) and nuclei counterstained with DAPI (blue) in EMOs in both monoculture and co‐culture conditions. Scale bars: 50 µm. Insets show higher‐magnification views of the structures. (I) Comparative immunofluorescence images of vimentin‐positive cells in EMOs cultured in monoculture and SPHEGaT co‐culture conditions.

Importantly, organoid expansion occurred in both conditions, and total organoid area did not differ between monoculture and co‐culture (Figure 2B). In contrast, architectural remodeling was strongly influenced by SPHEGaT exposure. The proportion of folded organoids increased from 2.39 ± 2.17% in monoculture to 13.69 ± 4.73% in co‐culture at day 6 (P < 0.001; Figure 2C), with progressive temporal accumulation observed only in the co‐culture condition. This morphological transition was further supported by a significant decrease in organoid circularity over time in the co‐culture condition, indicating increased structural complexity and folding, whereas circularity remained relatively stable in monoculture (Figure 2D). In addition, organoid solidity was significantly lower in co‐culture compared with monoculture, consistent with the development of a more irregular morphology characterized by epithelial invaginations and tissue remodeling (Figure S2). Thus, SPHEGaT‐derived cues promote structural reorganization rather than simple volumetric growth.

Optical‐density analysis further supported this morphogenic shift. Whereas monocultured EMOs maintained high translucency at later time points, co‐cultured organoids displayed a significant transition toward denser phenotypes by day 6 (Figure 2E), consistent with epithelial compaction and differentiation.

To determine whether remodeling was driven by altered proliferation, Ki67 immunostaining was performed. No significant differences in proliferative index were observed between conditions (Figure 2F,G), indicating that SPHEGaT exposure does not suppress or enhance epithelial proliferation in a sustained manner. Instead, architectural remodeling appears to reflect reorganization of epithelial structure rather than changes in cell cycle dynamics.

Immunostaining for E‐cadherin and vimentin revealed qualitative alterations in epithelial organization in SPHEGaT‐exposed EMOs (Figure 2H,I). Although vimentin intensity showed a consistent reduction across donor‐derived samples, this did not reach statistical significance (P = 0.1124), likely reflecting limited statistical power. Collectively, these results demonstrate that integrated ovarian signaling drives progressive epithelial morphogenesis independently of proliferative modulation.

### 2.4 SPHEGaT Co‐Culture Establishes a Hormone‐Responsive Differentiation State in EMOs

We next asked whether SPHEGaT‐induced architectural remodeling was accompanied by activation of hormone‐responsive differentiation programs. Estrogen receptor (ER) expression remained comparable between monocultures and co‐cultures. However, P4 receptor (PGR) expression was robustly increased in co‐cultured EMOs (Figure 3A). Whereas monocultured organoids showed low PGR positivity, with approximately 4.74 ± 3.56% of PGR‐positive nuclei, exposure to SPHEGaTs increased this proportion to approximately 40.54 ± 4.38% (Figure S2), indicating functional endocrine reprogramming.

**FIGURE 3:**
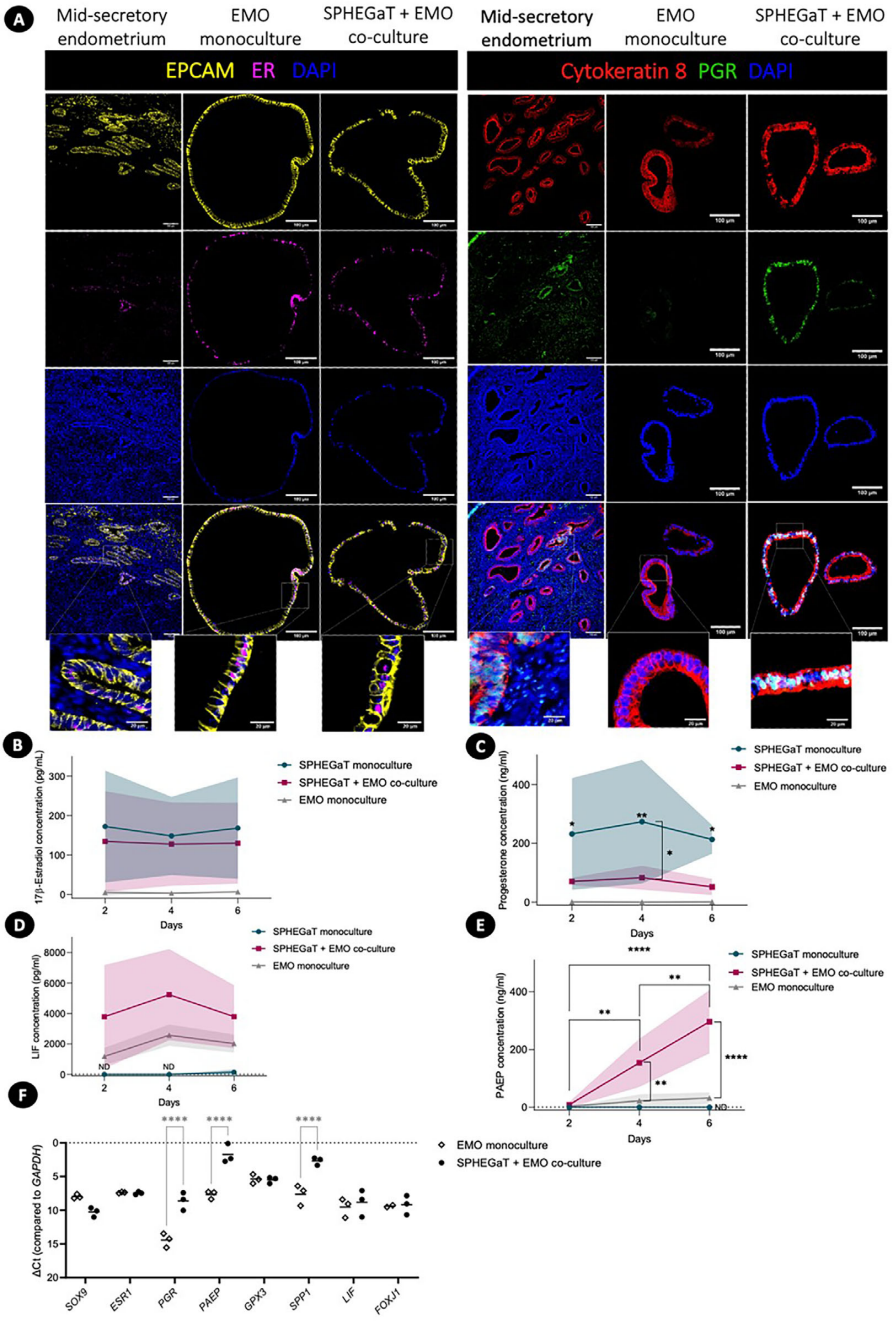
Secretory profile of the EMOs. (A) Representative immunofluorescence images of EMOs showing epithelial organization and hormone receptor expression. EPCAM (yellow), estrogen receptor (ER; magenta) and nuclei (DAPI; blue) are shown in the left panels. Cytokeratin 8 (red), progesterone receptor (PGR; green) and nuclei (DAPI; blue) are shown in the right panels. Insets show higher‐magnification views. Scale bars, 100 µm; inset scale bars, 20 µm. (B‐E) ELISA quantification of secreted 17β‐estradiol (E2), progesterone (P4), pregnancy‐associated endometrial protein (PAEP) and leukemia inhibitory factor (LIF) in culture media. Mean values are shown with shaded regions representing ± S.D. Magenta indicates SPHEGaT‐EMO co‐culture, turquoise indicates SPHEGaT monoculture, and grey indicates EMO monoculture. *n* = 3 biological replicates. Statistical analysis was performed using two‐way ANOVA followed by multiple comparisons testing. ^*^ *P* < 0.05, ^**^ P < 0.01, and ^**** ^P < 0.0001. (F) Quantitative RT‐qPCR analysis of gene expression in EMOs. Data are expressed as relative mRNA levels. Each doc represents a biological replicate. *n* = 3 independent biological replicates. Statistical significance was determined using two‐way ANOVA. ^****^ *P* < 0.0001.

Hormone quantification of culture media provided additional insight. Free E2 concentrations were comparable between SPHEGaT monocultures and co‐cultures (∼162.86 pg mL^−1^ vs ∼130.47 pg mL^−1^), while EMO monocultures produced negligible E2 (Figure 3B). In contrast, P4 levels were markedly reduced in co‐culture (∼68.45 ng mL^−1^) relative to SPHEGaT monoculture (∼239.43 ng mL^−1^) (Figure 3C), potentially reflecting progesterone metabolism, uptake, or other forms of ovarian‐endometrial interaction. It should be noted that the hormone concentrations reported in Figure 3 were measured after SPHEGaTs had completed the initial 9‐day assembly period and were subsequently maintained for an additional 2–6 days following alginate encapsulation under the co‐culture culture conditions. Consequently, these values are not directly comparable to those reported in Figure 1K, which were obtained at the end of spheroid formation prior to encapsulation. Because Figure 3 reflects the culture conditions used for ovarian‐endometrial communication experiments, these hormone concentrations represent the most relevant reference for interpreting the endocrine environment experienced by the EMOs. Differences in culture medium composition and alginate embedding may contribute to the distinct hormone concentrations observed between the two experimental settings.

Functional readouts of differentiation further supported endocrine activation. PAEP secretion increased progressively in cultures containing EMOs and was significantly enhanced in the SPHEGaT‐EMO co‐culture condition compared with EMO monoculture at corresponding time points (Figure 3D). LIF secretion exhibited donor‐dependent variability without statistical significance (Figure 3E).

At the transcriptional level, co‐cultured EMOs showed increased expression of P4‐responsive and secretory‐phase‐associated genes, including PAEP, SPP1, and HSD17B2 (Figure 3F), whereas SOX9, ESR1, GPX3, LIF, and FOXJ1 remained unchanged. Additional analysis showed that SOX17, GATA2, and AREG were expressed in EMOs but were not significantly altered by SPHEGaT co‐culture (Figures S2). Together, these findings indicate that SPHEGaT‐derived cues induce a selective P4‐responsive secretory state rather than a complete receptivity‐associated transcriptional program.

### 2.5 Static Hormone Supplementation Fails to Recapitulate SPHEGaT‐Driven Morphogenesis

To determine whether the observed differentiation depended solely on steroid concentration or on integrated ovarian signaling, we subjected SPHEGaT‐conditioned medium (SPHEGaT‐CM) to charcoal‐dextran (DCC) treatment to deplete endogenous steroids. DCC reduced E2 by ∼59% and P4 by ∼90% relative to untreated SPHEGaT‐CM (Figure 4A). Following steroid depletion, exogenous E2 and P4 were reintroduced to generate the SPHEGaT‐CM_DCC+E2+P4 condition.

**FIGURE 4:**
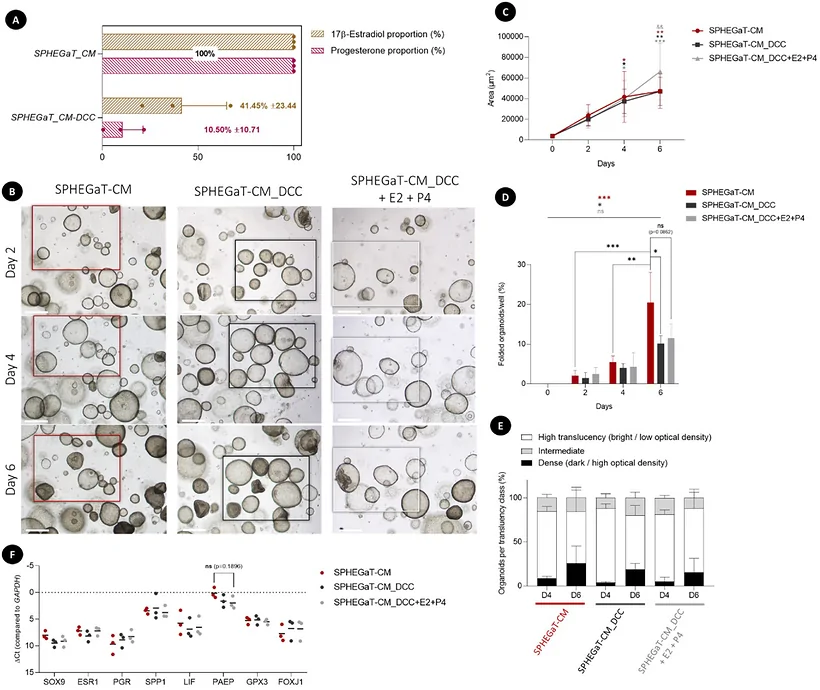
Characterization of EMO responses to SPHEGaT‐CM following charcoal‐dextran treatment and steroid supplementation. (A) Quantification of estradiol and progesterone content in SPHEGaT‐conditioned medium (SPHEGaT‐CM) before and after charcoal‐dextran (SPHEGaT‐CM_DCC) treatment. Hormone levels are expressed as the percentage of the concentration measured in untreated SPHEGaT‐CM (set to 100%). Data represent *n* = 3 independent biological replicates and are shown as mean ± S.D. (B) Representative brightfield images of organoids cultured for 2, 4, and 6 days in SPHEGaT‐CM, SPHEGaT‐CM_DCC, or SPHEGaT‐CM_DCC supplemented with exogenous E2 and P4. Images were acquired from independent fields using identical imaging settings. Colored boxes are included to improve visualization of organoid evolution over the days. Scale bar = 200 µm. (C) Quantification of total organoid area (µm^2^) over time under the three experimental conditions. Data represent mean ± SD from 3 independent biological replicates. Statistical analysis was performed using two‐way ANOVA by multiple comparisons test. * indicates a statistically significant difference relative to day 0 within the same condition; ^&^ indicates a statistically significant difference relative to SPHEGaT‐CM at the corresponding time point. ^*^P < 0.05, ^**^P < 0.01, ^***^P < 0.001; ^&&^P < 0.01. (D) Fold change in organoid number relative to day 0 for each experimental condition over the culture period. Data are presented as mean ± S.D. Data represent *n* = 3 independent biological replicates. Statistical analysis was performed using two‐way ANOVA by multiple comparisons test. ^*^P < 0.05, ^**^P < 0.01, ^***^P < 0.001, *n.s. = non‐significant*. (E) Distribution of organoids according to translucency class at days 4 and 6. Organoids were classified as high translucency (bright/low optical density), intermediate, or dense (dark/high optical density). Data are shown as percentage of total organoids per condition (mean ± S.D). Data represent *n*  = 3 independent biological replicates. (F) RT‐qPCR analysis of selected genes (*SOX9, ESR1, PGR, SPP1, LIF, PAEP, GPX3*, *FOXJ1, and HSD17B2*) in organoids cultured under the three experimental conditions. Gene expression is shown as *ΔCt* values (*Ct_target − Ct_reference*, *GAPDH*) and plotted on a reversed y‐axis, such that higher values correspond to higher expression. Each dot represents an independent biological replicate. Data represent *n* = 3 independent biological replicates. Statistical analysis was performed using two‐way ANOVA followed by multiple comparisons. *n.s. = non‐significant*.

Organoid growth, assessed by area, increased progressively in all conditions (Figure 4B,C). Notably, static hormone add‐back (SPHEGaT‐CM_DCC+E2+P4) resulted in significantly greater organoid expansion at day 6 compared with untreated SPHEGaT‐CM, whereas DCC treatment alone did not significantly alter growth relative to untreated CM.

Strikingly, the pattern of epithelial folding diverged from organoid expansion. Untreated SPHEGaT‐CM induced the greatest increase in folding over time, whereas DCC‐treated CM supported a reduced but still significant increase (Figure 4D and Figure S3). In contrast, static supplementation with exogenous hormones failed to restore folding and exhibited the lowest fold induction among conditions. At day 6, folding in untreated SPHEGaT‐CM remained significantly higher than in both DCC‐treated groups. Importantly, the CM assay differs substantially from the direct co‐culture system shown in Figure 2. Conditioned medium was collected every other day, resulting in intermittent rather than continuous exposure to SPHEGaT‐derived signals. Furthermore, differences in culture configuration may contribute to the distinct absolute folding percentages observed. Therefore, folding values between Figures 2 and 4 should not be directly compared.

Organoid translucency evolved similarly across conditions (Figure 4E), and RT‐qPCR analysis revealed no statistically significant differences in transcript levels among groups (Figure 4F), although PAEP expression trended downward in treated conditions.

Collectively, these findings demonstrate that epithelial morphogenesis cannot be reproduced by static hormone supplementation alone. Rather, the morphogenic response appears to depend on signaling components present within the SPHEGaT‐derived microenvironment that extend beyond steroid hormone exposure alone.

## 3 Discussion

### 3.1 Architecture as a Determinant of Endocrine Function

This study establishes that spatial organization of primary human GCs and stromal‐derived TLCs generates a viable and steroidogenically competent endocrine unit capable of instructing endometrial morphogenesis. Beyond demonstrating hormone production, our findings underscore a central principle: ovarian endocrine output is inseparable from 3D architecture.

Over the past decade, accumulating evidence has shown that 3D organization fundamentally alters GC transcriptional programs, cell‐cell coupling, and steroidogenic efficiency compared with 2D monolayers [20, 21, 22, 23]. Likewise, TCs exhibit biomechanical orientation and contractile behavior that regulate follicular mechanics and intercellular signaling, phenomena that cannot be recapitulated in planar systems [24]. Our multilayered spheroid recapitulates this compartmentalized arrangement, with GCs forming the core and TLCs constituting the outer layer, separated by a collagen interface that functionally mimics the follicular basement membrane. This configuration enables diffusion‐based metabolic coupling while preserving structural segregation.

Importantly, the fully human composition of SPHEGaT distinguishes it from prior ovarian constructs based on rodent cells or mixed‐species systems [14, 25]. The limited availability of primary human TCs has historically constrained translational ovarian engineering [26]. By leveraging stromal‐to‐theca differentiation protocols [27, 28], we generated TLCs expressing CYP17A1 and CD13, providing androgenic substrate to support granulosa aromatization. The resulting spheroids maintained high viability, exhibited minimal hypoxic burden, and produced sustained levels of E2 and P4, confirming their functional steroidogenic competence.

### 3.2 Integrated Ovarian Signaling Drives Epithelial Morphogenesis Rather Than Proliferation

Functional coupling of this ovarian unit to human endometrial organoids revealed that integrated endocrine signaling promotes epithelial remodeling independent of proliferative expansion. Although organoid size increased comparably in monoculture and co‐culture, SPHEGaT exposure selectively induced epithelial folding, densification, and architectural complexity.

The absence of significant differences in Ki67 expression suggests that structural remodeling is not driven by altered proliferative dynamics. This is consistent with in vivo physiology, where epithelial proliferation peaks during the estrogen‐dominant proliferative phase and stabilizes or declines during P4‐driven secretory maturation [29, 30]. The trend toward reduced vimentin expression in co‐culture further aligns with attenuation of epithelial‐mesenchymal plasticity observed during secretory differentiation [31]. The progressive decrease in both circularity and solidity observed in co‐cultured organoids further supports the occurrence of epithelial remodeling, indicating the emergence of increasingly irregular tissue architectures over time.

Notably, the degree of folding observed in co‐culture appeared greater than that commonly reported following static steroid supplementation in organoid systems, suggesting that ovarian‐derived cues provide instructive signals beyond hormone concentration alone. Together, these data indicate that SPHEGaT‐derived signaling promotes epithelial morphogenesis through mechanisms that cannot be explained by E2‐dependent proliferation alone.

### 3.3 Context‐Dependent P4 Signaling Within a Coupled Endocrine Axis

At the molecular level, SPHEGaT co‐culture induced robust PGR expression and upregulation of P4‐responsive and secretory‐phase‐associated genes, including PAEP, SPP1, and HSD17B2. These changes establish a P4‐responsive epithelial state consistent with secretory‐phase differentiation, without evidence of complete receptivity‐associated differentiation or lineage switching.

A particularly notable observation was the selective reduction of P4 levels in co‐culture relative to SPHEGaT monoculture, whereas E2 levels remained stable. This hormone‐specific modulation suggests that the endometrium may influence ovarian steroid dynamics within the coupled system. In vivo, uterine‐derived prostaglandins and other mediators are known to regulate luteal P4 output [32], supporting the plausibility of reciprocal communication. Although direct modulation of SPHEGaT steroidogenic enzymes was not assessed here, the selective alteration in P4 concentration highlights the capacity of this model to capture bidirectional endocrine features.

Thus, the co‐culture system does not merely expose endometrial tissue to exogenous hormones; it establishes a coupled endocrine axis in which hormone availability reflects ongoing integration between compartments.

### 3.4 Dynamic Integration Versus Static Hormone Supplementation

To disentangle steroidal from non‐steroidal contributions, we implemented steroid depletion and add‐back experiments. Strikingly, near‐complete depletion of P4 did not abolish epithelial folding, whereas static supplementation with exogenous E2 and P4 failed to restore the morphogenic phenotype observed with intact SPHEGaT‐conditioned medium.

These findings suggest that epithelial morphogenesis cannot be explained by hormone concentration alone and may depend on additional components of the SPHEGaT‐derived signaling environment. Static add‐back likely fails to reproduce temporal fluctuations, concentration gradients, local metabolism, carrier proteins, and co‐secreted paracrine factors inherent to an intact ovarian unit. Moreover, supraphysiologic or imbalanced steroid exposure may bias organoids toward expansion rather than differentiation, potentially contributing to the increased area and reduced folding observed following hormone supplementation. While E2 and P4 are major regulators of endometrial function, the ovarian follicle functions as a highly complex endocrine and paracrine unit that produces a broad repertoire of bioactive signals in addition to steroid hormones. Such signals may include ovarian hormones of the inhibin/activin family, as well as growth factors, cytokines, metabolites, carrier proteins, and extracellular vesicle (EV)‐associated cargo generated through interactions between granulosa and TC compartments [33, 34, 35, 36]. In particular, inhibins and activins have been implicated in endometrial remodeling, receptivity, and implantation‐associated processes, suggesting that they may contribute to the coordinated epithelial responses observed in the present model [37, 38]. Likewise, EVs released by ovarian somatic cells have emerged as important mediators of intercellular communication through the transfer of proteins, lipids, mRNAs, and microRNAs capable of modulating the behavior of recipient tissues [34, 36]. Furthermore, factors such as bFGF and EGF‐like ligands have been reported to be produced by ovarian somatic cells and are recognized regulators of endometrial epithelial proliferation and differentiation, suggesting that they may interact with steroid signaling pathways and influence tissue remodeling [39, 40, 41, 42]. Importantly, EGF and bFGF were present in both EMO monocultures and SPHEGaT co‐cultures. Therefore, although these factors may contribute to epithelial behavior, their presence alone is insufficient to explain the morphogenic and differentiation responses observed following SPHEGaT exposure.

The directional reduction of PAEP expression after steroid add‐back further supports the notion that secretory differentiation requires coordinated signaling rather than isolated hormone exposure. Together, these results highlight limitations of hormone‐only models and indicate that the morphogenic response cannot be fully recapitulated by static steroid supplementation alone.

### 3.5 Implications and Future Directions

By establishing a human, spatially organized ovarian‐endometrial platform, this study provides a tractable system to investigate integrated endocrine regulation and tissue communication. The current configuration models a P4‐dominant environment; future incorporation of a sequential E2‐priming phase followed by SPHEGaT exposure could reproduce the full proliferative‐to‐secretory transition of the menstrual cycle. Future studies should also further characterize the cellular organization of the remodeled epithelial structures induced by SPHEGaT exposure. Although the folded regions retained expression of EPCAM, cytokeratin 8, and E‐cadherin, additional analyses will be required to determine whether these structures exhibit epithelial stratification, altered polarity, or the emergence of distinct epithelial subpopulations.

Beyond physiological modeling, this platform offers opportunities to explore P4 resistance, implantation failure, endocrine disruption, and therapeutic ovarian constructs. More broadly, the findings suggest that endocrine signaling in reproductive tissues is fundamentally context‐dependent and cannot be faithfully recapitulated through static hormone replacement alone.

Despite its strengths, several limitations should be acknowledged. First, the system models a P4‐dominant endocrine environment without a preceding E2‐priming phase and therefore does not fully recapitulate the complete proliferative‐to‐secretory transition of the menstrual cycle. This may contribute to the selective transcriptional response observed in co‐cultured EMOs, in which PAEP, SPP1, and HSD17B2 were increased, whereas LIF, AREG, SOX17, and GATA2 were not significantly altered, suggesting activation of a partial P4‐responsive secretory program rather than full acquisition of an endometrial receptive state. Indeed, LIF induction has been shown to depend on proper E2‐priming before P4 exposure [43]. Moreover, full activation of receptivity‐associated pathways likely requires additional multicellular crosstalk involving stromal decidualization, immune components, and embryo‐derived signals [44, 45]. Thus, while the current epithelial‐only co‐culture captures selected features of P4‐responsive secretory differentiation, it does not fully reproduce the complete implantation‐window program. Second, although P4 dynamics were selectively altered in co‐culture, direct assessment of steroidogenic enzyme expression within SPHEGaTs after coupling was not performed, limiting mechanistic resolution of potential feedback regulation. Third, steroid depletion using charcoal‐dextran achieved substantial but incomplete hormone reduction, and residual steroid activity may have contributed to epithelial responses. In addition, although conditioned media were centrifuged and filtered following charcoal‐dextran treatment, we cannot completely exclude the possibility that charcoal‐dextran treatment altered bioactive components beyond steroid hormones or that non‐steroidal factors contributed to the observed outcomes. Therefore, the conditioned‐medium experiments should be interpreted as evaluating the effects of hormone depletion within a complex ovarian secretome rather than as a selective manipulation of steroid signaling alone.

Furthermore, the CM experiments did not include a hormone‐supplemented basal medium control. Therefore, while static E2 and P4 supplementation failed to reproduce the effects of intact SPHEGaT‐CM, the relative contribution of dynamically generated endocrine signaling versus non‐steroidal SPHEGaT‐derived factors could not be determined. Finally, the study focuses on epithelial remodeling and does not incorporate stromal decidualization, immune components, vascular elements, or trophoblast interactions, all of which contribute to in vivo endometrial physiology.

An additional limitation of this study is the use of TLCs generated from ovarian stromal cells isolated from postmenopausal donors, whereas granulosa cells were obtained from reproductive‐age women. Nevertheless, our previously validated differentiation protocol demonstrated that these cells acquire key theca‐like characteristics, including expression of CYP17A1, CD13, STAR, PLIN2, and IGF1, together with the production of androstenedione, progesterone, and DHEA [28]. However, we cannot exclude the possibility that age‐related molecular or epigenetic features persist even after differentiation. Further studies will be required to determine the extent to which donor age influences TLC phenotype and function.

These constraints define clear avenues for future refinement while not diminishing the central conclusion that our ovarian construct provides an endogenous and functionally active source of endocrine signaling. Collectively, these findings demonstrate that ovarian‐endometrial communication is not dictated solely by hormone concentration, but is influenced by additional components of the ovarian‐derived microenvironment. By reconstructing a multilayered human ovarian unit and coupling it to endometrial organoids, we show that SPHEGaTs qualitatively influence hormone bioactivity and epithelial morphogenesis. Static hormone supplementation failed to reproduce these effects, indicating that physiological ovarian‐endometrial communication cannot be fully recapitulated by isolated hormone exposure alone. This study therefore highlights the importance of integrated ovarian‐derived signaling in reproductive endocrine function and provides a new framework for modeling human ovarian‐endometrial interactions in vitro.

## 4 Conclusion

The human ovarian‐endometrial platform described here provides a controllable framework for reconstructing endocrine communication in vitro. By functionally coupling engineered ovarian spheroids with endometrial organoids, this system enables investigation of ovarian‐derived signaling in a human‐specific setting and creates opportunities to study fertility disorders, endocrine disruption, and ovarian bioengineering strategies. More broadly, it establishes a foundation for next‐generation reproductive models that move beyond hormone supplementation toward the reconstruction of tissue‐level endocrine function.

## 5 Experimental Section

### 5.1 Human Ovarian Tissue and Stromal Cell Isolation

Whole ovaries were obtained from postmenopausal multiorgan donors (Table S1) aged 49–58 years (mean± S.D.: 53.8 ± 4.02 years). The use of the human ovarian tissue was approved by the Institutional Review Board (IRB) of Université Catholique de Louvain on 23 May 2019 (IRB reference 2018/19DEC/475). Following procurement, ovaries were transported to the laboratory in Dulbecco's phosphate‐buffered saline (DPBS, Gibco, MA, USA, Cat#14190144) at 4°C. Upon arrival, the medullary region was removed, after which the cortical tissue was cut into approximately 5 × 5 × 1 mm fragment and frozen following a standard procedure [46].

For cell isolation, thawed cortical fragments were enzymatically digested using 0.28 U mL^−1^ Liberase‐DH (Sigma‐Aldrich, St.Louis, MO, USA, Cat# 5401054001) and 8 KU mL^−1^ DNase I (Sigma‐Aldrich, Cat# D4263) at 37°C for 75 min with gentle agitation and intermittent pipetting, as previously described [47]. Enzymatic activity was quenched by adding an equal volume of DPBS supplemented with 10% fetal bovine serum (FBS; Gibco, Cat# 16140‐071). The resulting cell suspension was sequentially filtered through 80 µm (Sigma–Aldrich, Cat#NY8004700) and 20 µm (Sigma‐Aldrich, Cat# NY2004700) nylon mesh filters to remove undigested tissue. Cells were centrifuged, counted, and allocated for downstream differentiation into TLCs.

### 5.2 Differentiation of Ovarian Stromal Cells Into TLCs

Differentiation of stroma cells into TLCs was performed following the protocol described by Asiabi et al. [27], with minor adaptations. Briefly, isolated ovarian stromal cells were counted and seeded onto collagen type I‐coated culture dishes (StemCell, Cologne, Germany, Cat# 100–0364) at a density of 70,000 cells mL^−1^. Cells were cultured in TC differentiation medium consisting of DMEM/F12 supplemented with GlutaMAX (Gibco, Cat# 31331‐093), 10% KnockOut Serum Replacement (KSR; Gibco, Cat#10828‐010), 1% antibiotic‐antimycotic solution (Anti‐Anti; Gibco, Cat# 15240‐062), 1% insulin‐selenium‐transferrin (ITS; Gibco, Cat# 41400‐045), 100 ng mL^−1^ stem cell factor (SCF; PeproTech, NJ, USA, Cat# 300–07), 20 ng mL^−1^ bone morphogenetic protein 6 (BMP‐6; PeproTech, Cat# 120‐06), 20 ng mL^−1^ recombinant human transforming growth factor beta 1 (TGF‐β1; PeproTech, Cat# 100–21C), 20 ng mL^−1^ recombinant human hepatocyte growth factor (HGF; PeproTech, Cat# 100–39H), 20 ng mL^−1^ recombinant human keratinocyte growth factor (KGF; PeproTech, Cat# 100–19), 100 ng mL^−1^ recombinant human insulin‐like growth factor 1 (IGF‐1; PeproTech, Cat# 100–11), 10 ng mL^−1^ recombinant human basic fibroblast growth factor (bFGF; PeproTech, Cat# AF‐100‐18B), 20 ng mL^−1^ recombinant human epidermal growth factor (EGF; PeproTech, Cat# AF‐100‐15), 20 ng mL^−1^ recombinant human growth differentiation factor 9 (GDF‐9; Bio‐Techne, Dublin, Ireland, Cat# 8266‐G9‐010) and 7.06 mIU mL^−1^ Menopur (Ferring, Aalst, Belgium).

Cells were maintained for 7 days at 37°C in a humidified atmosphere containing 5% CO_2_, with half of the medium replaced every other day. At the end of the differentiation period, TLCs were collected and allocated for spheroid's formation and immunofluorescence analyses.

### 5.3 GC Collection

The use of GCs was approved by the IRB of Université Catholique de Louvain on 3 June 2019 (IRB reference 2014/16DEC/597). Inclusion criteria comprised women undergoing in vitro fertilization (IVF) or intracytoplasmic sperm injection (ICSI) for infertility treatment, as well as patients undergoing oocyte vitrification for fertility preservation. Donors (Table S2) were between 24 and 41 years of age (mean± SD: 35.5± 4.36 years). Cumulus‐oocyte complexes were obtained during routine oocyte retrieval procedures at the IVF Department of the Cliniques Universitaires Saint‐Luc. For clinical purposes, cumulus/GCs were enzymatically removed using HYASE‐10X (Vitrolife, Londerzeel, Belgium, Cat#26284), followed by gentle mechanical dissociation to ensure complete separation of GCs from the oocytes. The isolated GCs were washed twice in pre‐warmed Sperm Preparation Medium (Origio, Måløv, Denmark, Cat#10700060) to remove residual enzymatic activity.

Following processing, oocytes were immediately returned to the embryology laboratory for assisted reproductive procedures, while the GCs were collected and transported to the research laboratory in the same medium for subsequent spheroids formation and immunofluorescence analyses.

### 5.4 Multilayered Spheroid Formation

Multilayered spheroids comprising GCs and TLCs, hereafter referred to as SPHEGaTs, were generated using a stepwise 3D assembly strategy. Primary GCs (2,000 cells per spheroid), pooled from 2–5 patients on each collection day, were first seeded into 96‐well ultra‐low attachment plates (Sarstedt, Nümbrecht, Germany, Cat# 83.3925.400). Each biological replicate corresponded to an independent collection day and was therefore generated from a distinct GC pool. GCs spontaneously aggregated into compact 3D spheroids within 48 h in SPHEGaT medium. The medium consisted of DMEM/F12 (Gibco, Paisley, UK, Cat#21041‐025) supplemented with 5% KRS, 1% Anti‐Anti, 1% ITS, 2% L‐glutamine (Thermo Fisher Scientific, Cat#25030‐024), 100 ng mL^−1^ IGF‐1, 1 mIU mL^−1^ Menopur, 499 mIU mL^−1^ Puregon (Organon, New Jersey, USA), 10 ng mL^−1^ EGF, 10 ng mL^−1^ bFGF, 3.62 µg mL^−1^ hydrocortisone (Sigma‐Aldrich, Cat# H0888‐1G), 20 µg mL^−1^ cholesterol (Sigma‐Aldrich, Cat# C3045‐5G), 2 ng mL^−1^ TGF‐β1, 10 ng mL^−1^ leukemia inhibitory factor (LIF; PeproTech, Cat# 300–05), 20 ng mL^−1^ BMP‐6, 20 ng mL^−1^ GDF‐9, 50 ng mL^−1^ SCF, 20 ng mL^−1^ HGF, and 0.1% human serum albumin (HSA; CSL Behring, Berne, Switzerland).

After 48 h, a 0.001% type I collagen solution (Serva, Heidelberg, Germany, Cat# 47256) was added to deposit a thin collagen layer around the GC spheroid surface. Spheroids were maintained in this collagen‐supplemented environment for an additional 24 h. Inclusion of this collagen coating was incorporated to better mimic the basement membrane found in the human follicle.

Subsequently, TLCs (2,000 cells) were added to the wells, enabling them to organize around the collagen‐coated GC core and establish a multilayered follicle‐like structure. The assembled SPHEGaTs were cultured for an additional 6 days at 37°C in a humidified atmosphere containing 5% CO_2_, with half of the medium refreshed every other day.

### 5.5 Organoid Culture from Endometrial Biopsies

Endometrial biopsies (Table S3) were obtained from women aged 28–30 years (mean± SD: 29.33 ± 1.15 years). The study was approved by the UZ Leuven Ethical Committee (S59006 and S65570), and all samples were collected after obtaining written informed consent from all participants.

Biopsies were transported to the laboratory in cold medium, washed extensively, minced and enzymatically digested with collagenase IV (1 mg mL^−1^; Life Technologies) for 20–40 min at 37°C. This was followed by mechanical dissociation using a fire‐polished Pasteur pipette, resulting in a heterogeneous cell suspension, as previously described [10].

After centrifugation, the cell pellet was resuspended in 70% Matrigel (Corning, NY, USA, Cat# 356231) supplemented with Rock inhibitor (RI, Y‐27632, STEM CELL Technologies, BC, Canada, Cat# 72304), and 20 µL domes were plated onto pre‐warmed culture plates. Domes were allowed to polymerize for 20–30 min at 37°C before adding EMO medium. The EMO medium consisted of DMEM/F12 supplemented (Gibco, Cat# 31330‐038) with 1× penicillin‐streptomycin (Gibco, Cat# 15140122), 1× L‐glutamine, 1× ITS, 1× N2 supplement (Gibco, Cat# 17502048), 1× B27 supplement without vitamin A (Gibco, Cat# 12587010), 5 mM nicotinamide (Sigma‐Aldrich, Cat# N0636), 1.25 mM N‐acetyl‐L‐cysteine (Sigma‐Aldrich, Cat# A7250), 0.5 µM A83‐01 (Sigma‐Aldrich, Cat# SML0788), 2 ng mL^−1^ bFGF (R&D Systems, MN, USA, Cat# 234‐FSE‐025), 50 ng mL^−1^ EGF (R&D Systems, Cat# 236‐EG‐200), 10 ng mL^−1^ recombinant human fibroblast growth factor 10 (FGF10; Peprotech, Cat# 100–26), 1 µM p38 MAPK inhibitor SB202190 (Tocris, Bristol, UK, Cat#1264), 10% R‐spondin‐1 conditioned medium (CM), and 10% Noggin CM.

Organoids were maintained at 37°C in a humidified atmosphere containing 5% CO_2_, with medium refreshed every two days. For passaging, Matrigel domes were disrupted using cold DMEM/F12, followed by incubation with TrypLE Express (Gibco, Cat# 12605010) for 5 min at 37°C and strong pipetting. After centrifugation, cells were resuspended in DMEM/F12 and re‐embedded in 70% of Matrigel with RI to generate new domes. EMOs were passaged every 7–10 days at an approximate split ratio of 1:4. All experiments were performed using organoids between passage 3 and passage 8.

### 5.6 Mid‐Secretory Phase Human Endometrial Tissue

Mid‐secretory phase human endometrial formalin‐fixed, paraffin‐embedded (FFPE) sections were kindly provided by a colleague from the De Duve Institute (UCLouvain), with appropriate ethical approval and informed consent. The tissue served as a physiological reference control for immunofluorescence analyses.

### 5.7 SPHEGaT‐EMO Co‐Culture System

For the establishment of the SPHEGaT‐EMO co‐culture system, three‐day old intact EMOs were first recovered from Matrigel domes by incubation with cold cell recovery solution (Corning, Cat# 354253) for 30 min at 4°C, followed by centrifugation and multiple wash steps. Pipet tips and Eppendorf tubes were precoated with 3% BSA to prevent organoids adhering to the plastic. The resulting pellet was resuspended in DMEM/F12 and 70% Matrigel supplemented with RI. This mixture was plated into pre‐warmed 24‐well culture plates. Matrigel was allowed to polymerize for 25–30 min at 37°C in a humidified incubator containing 5% CO_2_.

In parallel, a 1% (w/v) alginate (Sigma–Aldrich, Cat# A2033‐100G) solution was prepared, and SPHEGaTs were embedded at a density of two spheroids per 15 µL alginate droplet. Alginate droplets were ionically crosslinked by incubation with 100 mM CaCl_2_ (Sigma‐Aldrich, Cat# C7902) for 10 min. Following crosslinking, two alginate droplets, each containing two SPHEGaTs, were transferred into wells containing the pre‐embedded EMOs cultures.

Control conditions were established in parallel. EMO monocultures were generated by culturing Matrigel‐embedded organoids in the presence of empty alginate droplets, while SPHEGaT monocultures were maintained by culturing alginate‐embedded spheroids in the presence of empty Matrigel domes. These control groups were maintained under identical conditions and using the same co‐culture medium as the experimental co‐cultures.

Cultures were maintained in co‐culture medium consisting of DMEM/F12 (Gibco, Cat# 21041‐025) supplemented with 1% KRS, 1% Anti‐Anti, 1% ITS, 2% L‐glutamine, 1× N2 supplement, 1× B27 supplement without vitamin A, 0.1% HSA, 100 ng mL^−1^ IGF‐1, 10 mIU mL^−1^ Menopur, 490 mIU mL^−1^ Puregon, 50 ng mL^−1^ EGF (PeproTech, AF‐100‐15), 10 ng mL^−1^ bFGF (PeproTech, AF‐100‐18B), 0.5 mM N‐acetyl‐L‐cysteine, 2.5 mm nicotinamide, 100 ng mL^−1^ recombinant human R‐spondin‐1 (Peprotech, Cat#120‐38), 25 ng mL^−1^ recombinant human Noggin (PeproTech, Cat# 120‐10C), and 0.5 µm A83‐01. SPHEGaT‐EMO co‐cultures were maintained for 6 days at 37°C in a humidified atmosphere containing 5% CO_2_, and culture medium was refreshed and collected every other day for downstream analyses.

### 5.8 Hormonal Validation Using SPHEGaT‐Conditioned Media

To assess the effects of SGHEGaT‐derived soluble factors on EMOs, spheroid‐conditioned medium (CM) was generated and subjected to steroid hormone depletion prior to treatment of EMOs. SPHEGaTs were embedded in alginate at a density of two spheroids per 15 µL alginate droplet, ionically crosslinked as described above, and cultured at two droplets per well. Spheroids were maintained for 6 days at 37°C in 5% CO_2_, and CM was collected every two days. One‐fifth of the CM was retained as untreated control (SPHEGaT‐CM). The remaining CM was processed using dextran‐coated charcoal (DCC) to remove endogenous steroid hormones. DCC resin (Sigma‐Aldrich, Cat# C6241SG) was added at a final concentration of 0.15% (w/v) and incubated for 15 min at 4°C with gentle agitation. The suspension was centrifuged at 10,000 × g for 10 min, and the supernatant was filtered through a 0.22 µm membrane and stored at −80°C until use. As expected for single‐round DCC treatment, depletion was substantial but incomplete, reducing E2 by ∼59% and P4 by ∼90%. This partial depletion is consistent with established DCC efficiency and was considered when interpreting downstream responses.

For treatment experiments, EMOs were recovered from Matrigel as previously described, replated in 20 µL domes in pre‐warmed 48‐well plates, and cultured with one of the following conditions: *(1) SPHEGaT‐CM alone (untreated CM), (2) SPHEGaT‐CM_DCC (hormone‐depleted CM) and (3) SPHEGaT‐CM_DCC + E2 [300 pg mL^−^^1^(Sigma–Aldrich, Cat#E2758)] + P4 [500 ng mL^−^^1^(Sigma–Aldrich, Cat#P7556)]*. These concentrations were selected to ensure effective hormone replacement following charcoal‐dextran treatment and to avoid hormone availability becoming a limiting factor in the add‐back condition. Although they exceeded the average hormone concentrations measured in SPHEGaT monocultures, they remained within the same order of magnitude and were intended to restore a hormone‐rich signaling environment.

Organoids were cultured for 6 days at 37°C in 5% CO_2_. Medium was refreshed every two days, with freshly prepared E2 and/or P4 added at each medium change.

### 5.9 Morphological Analysis

Bright‐field Images of SPHEGaT were acquired at days 3 and 6 using an inverted microscope (Leica DMIL, Diegem, Belgium). Spheroid area, circularity and solidity was quantified in ImageJ software following spatial calibration, with each spheroid segmented into an individual region of interest (ROI). Circularity values range from 0 to 1, with values closer to 1 indicating a more circular morphology and lower values reflecting increased folding and structural complexity. Solidity represents the ratio between the organoid area and the area of its convex hull, with lower values indicating greater structural irregularity and the presence of invaginations.

For co‐culture experiments, EMOs were imaged at days 0, 2, 4, and 6. Organoid area were quantified using ImageJ. EMO folding was assessed manually based on the presence of folded or invaginated epithelial structures. Translucency was quantified by mean pixel intensity (MIP) and classified as dense (MIP < 140), intermediate (140 ‐165), or high translucency (>165).

### 5.10 Immunofluorescence Analyses

#### 5.10.1 Cell Tracker Labelling

For selected experiments, TLCs were labeled prior to SPHEGaT assembly using a red fluorescent cell tracker dye (Vybrant^TM^ DiO; Thermo Fisher Scientific, Cat#V22886) according to the manufacturer's instructions. Labeled TLCs were incorporated into spheroids and cultured under standard conditions. Before co‐culture, fluorescent spheroids were fixed in 4% paraformaldehyde (PFA; Thermo Fisher Scientific, Cat#J61899) for 1h at room temperature (RT), permeabilized with Tween‐20 (VWR, Cat# 663684B), counterstained with Fluoroshield containing DAPI (Sigma‐Aldrich, St. Louis, USA, Cat#F6057), and imaged as z‐stacks using a confocal laser‐scanning microscope (LSM 800; Carl Zeiss Microscopy GmbH, Jena, Germany).

#### 5.10.2 Standard Immunofluorescence

Immunofluorescence analyses were performed on adherent cells, spheroids, and organoids using sample‐adapted protocols. For adherent cell staining, GCs and TLCs were plated onto coated dishes, allowed to adhere for 20 min at 37°C, fixed with 4% PFA for 20 min at RT, permeabilized with Tween‐20, and blocked with normal goat serum (NGS; Sigma‐Aldrich, Cat# S26). Samples were incubated overnight at 4°C with primary antibodies against CYP19A1, CYP17A1, CD13, and FSHR, followed by fluorophore‐conjugated secondary antibodies and Hoechst counterstaining (Invitrogen, Cat# H3570). See antibodies’ information in Table S4.

For SPHEGaT analysis, spheroids were fixed in 4% PFA for 1h at RT, embedded in agar, and processed for paraffin embedding. Sections (5 µm) were mounted on adhesion slides, quenched with 50 mm NH_4_Cl, and subjected to citrate‐based antigen retrieval. After permeabilization using Tween‐20 and blocking [NGS‐bovine serum albumin (BSA; Sigma‐Aldrich, Cat# A7030)], sections were incubated overnight incubation at 4°C with anti‐collagen type I. Negative controls omitted the primary antibody; human placental villi were used as positive controls.

For EMOs analysis, samples collected after co‐culture were fixed in 4% PFA for 45 min at RT, embedded in agar, and paraffin embedded. Sections (3 µm) underwent citrate antigen retrieval, permeabilization using Tween‐20 and NGS‐BSA blocking. Primary antibodies included Ki67, ER, PGR, cytokeratin 8 (CK8), EPCAM, vimentin, and E‐cadherin (E‐Cad), see Table S4. Negative controls consisted of sections processed without primary antibodies, while human endometrial tissue in the secretory phase served as positive controls.

After incubation with the appropriate fluorophore‐conjugated secondary antibodies for 45 min at RT, slides were mounted using Fluoroshield containing DAPI for nuclear counterstaining and imaged using a widefield fluorescence microscope (Eclipse Ni‐L; Nikon Instruments Europe B.V., Amsterdam, The Netherlands). Quantification was performed using ImageJ software (v1.54q).

### 5.11 Light‐Sheet Microscopy

SPHEGaTs were collected prior to co‐culture, fixed in 4% PFA for 24 h at RT and permeabilized/ blocked overnight at 4°C in Triton X‐100 (Sigma‐Aldrich, Cat# 93420) and BSA. Whole‐mount immunostaining was performed using primary antibodies against HIF‐2α and collagen type I (Table S4) diluted in Triton/BSA‐containing buffer. Spheroids were incubated with primary antibodies for 5 days at 4°C, followed by incubation with the corresponding fluorophore‐conjugated secondary antibodies together with a Hoechst as nuclear counterstain for 4 days at 4°C. Samples were post‐fixed in 4% PFA for 2 h at RT and cleared using a CUBIC‐based clearing protocol [25 wt.% urea (Sigma‐Aldrich, Cat# U1250), 50 wt.% sucrose (Sigma‐Aldrich, Cat# S9378), 10 wt.% triethanolamine (Sigma‐Aldrich, Cat# 90279), and 0.05% Triton X‐100] for 2 days at RT. Cleared SPHEGaTs were imaged using a light‐sheet fluorescence microscope (LighSheet Z.1; Carl Zeiss Microscopy GmbH, Jena, Germany), and 3D reconstructions were generated in IMARIS software (version 9.8.2).

### 5.12 Live/Dead Viability Assay

After 6 days of culture, SPHEGaT viability was assessed using a Live/Dead assay (Molecular Probes, Leiden, The Netherlands). Spheroids were incubated with 2 µm calcein‐AM and 5 µm ethidium homodimer‐1 in PBS for 45 min at 37°C in 5% CO_2_. Imaging was performed using both inverted fluorescence (Leica DMIL, Diegem, Belgium) and confocal laser‐scanning microscope (LSM 800; Carl Zeiss Microscopy GmbH, Jena, Germany). Image acquisition and processing were performed using ZEN Blue software (v3.10), and quantitative analysis was conducted in ImageJ. By defining ROIs and calculating the relative area of calcein (live) versus ethidium homodimer (dead) signal.

### 5.13 Gene Expression Analysis by RT‐qPCR

EMOs from co‐culture and CM‐treatment experiments were recovered by removing alginate droplets, dissolving Matrigel in ice‐cold DMEM/F12, and centrifuging the organoids. Total RNA was extracted using the RNeasy Micro Kit (Qiagen, Hilden, Germany, Cat# 74004) according to the manufacturer's instructions. Complementary DNA (cDNA) synthesis was performed using the iScript cDNA synthesis kit (Bio‐Rad Laboratories, MA, USA, Cat# 1708891). Quantitative real‐time PCR (qPCR) was performed using SYBR Green chemistry (Platinum SYBR Green qPCR SuperMix‐UDG; Invitrogen, MA, USA, Cat#11733046) on a StepOnePlus Real‐Time PCR System (Applied Biosystems, Waltham, MA, USA).

Gene expression analysis was performed for *SOX9*, *ESR1*, *PGR, SPP1*, *LIF*, *PAEP*, *GPX3*, *FOXJ1, HSD17B2, SOX17, AREG, and GATA2*. Primer sequences (forward and reverse) are listed in Table S5. *GAPDH* was used as housekeeping gene. Relative expression between experimental conditions and reference samples was calculated using the ΔCt method.

### 5.14 Hormone Secretion Analysis

All media were centrifuged to remove debris and stored at −80°C until analysis. E2 and P4 concentrations were measured in SPHEGaT media, untreated and DCC‐treated CM, and co‐culture media at days 2, 4, and 6 using commercially available ELISA kits: human 17β‐Estradiol (ADI‐900‐008, Enzo, New York, NY, USA) and human P4 (Peachtree Corners, USA, RayBiotech, Cat# 126EIA‐P4). PAEP and LIF levels were quantified in co‐culture media at the same timepoints using human PAEP ELISA kit (Peachtree Corners, USA, RayBiotech, Cat# ELH‐PP14) and human LIF ELISA kit (Dublin, Ireland, Bio‐Techn, Cat# DLF00B). All measurements were performed according to the manufacturers’ instructions, and samples and standards analysed in duplicate.

### 5.15 Statistical Analysis

All statistical analyses were performed using GraphPad Prism (v10). Data are presented as mean ± standard deviation (SD) unless otherwise indicated. Each biological replicate represents an independent donor‐derived cell preparation or organoid line. Technical replicates were averaged prior to statistical testing.

Normality of data distribution was assessed using the Shapiro‐Wilk test. For comparisons between two groups, unpaired two‐tailed Student's t‐tests were applied when data met parametric assumptions; otherwise, non‐parametric equivalents were used. For experiments involving multiple groups or timepoints, one‐ or two‐way ANOVA was performed as appropriate, followed by Tukey's or Sidak's post hoc correction for multiple comparisons. Paired analyses were used when repeated measurements were obtained from the same donor‐derived samples.

A two‐sided p‐value < 0.05 was considered statistically significant.

## Author Contributions

M.J.S.: conceptualization, methodology, validation, formal analysis and writing; S.D.V.: methodology, conceptualization and validation; L.L.: methodology; T.F.R.R.: methodology and validation; H.V.: conceptualization, resources, review and supervision; C.A.A.: conceptualization, resources, review, supervision, project administration, and funding acquisition.

## Funding

This study was supported by grants from the UCLouvain's Fonds spéciaux de recherche (IREC FSR22, awarded to C.A.A., Ph.D. scholarship awarded to M.J.S.), the Fonds National de la Recherche Scientifique (2025/V 6/5/027 – JG/DeM – 1698, awarded to C.A.A., scholarship awarded to T.F.R.R., and J.0003.26, awarded to C.A.A.), S.D.V (1S00825N) was supported by a PhD Fellowship from the Fonds voor Wetenschappelijk Onderzoek (FWO) Vlaanderen, and L.L (GPUCL/23/005) was supported by Bijzonder Onderzoeksfonds KU Leuven.

## Conflicts of Interest

The authors declare no conflicts of interest.

## Supporting information




**Supporting File 1**: advs76538‐sup‐0001‐SuppMat.docx.

**Supporting File 2**: advs76538‐sup‐0002‐Video S1_HIF2a Video.mp4.

## Data Availability

The data that support the findings of this study are available in the supplementary material of this article.
