# Supplementary material for: Spatially Organized Human Ovarian Spheroids Instruct Endometrial Morphogenesis
Source: Adv Sci (Weinh). 2026 Jul 9;13(55):e76538. doi: 10.1002/advs.76538 (PMC13348657; doi:10.1002/advs.76538)
Supplement: Supplementary file 1 [file ADVS-13-e76538-s002.docx]

**Spatially Organized Human Ovarian Spheroids Instruct Endometrial Morphogenesis**

Maria João Sousa, Silke De Vriendt, Lixian Liu, Thalles Fernando Rocha Ruiz, Hugo Vankelecom, Christiani Andrade Amorim^*^

**Figure S1. Effect of collagen coating on multilayered spheroid steroidogenic function. (A)** Schematic representation of multilayered spheroids with or without collagen coating. **(B)** E2 secretion measured by ELISA over 27 days from alginate-embedded spheroids cultured in multilayered spheroid medium. Three spheroids per condition were embedded in 15 μL alginate droplets. SPHEGaTs are shown in magenta and TG-Layered Spheroids in blue. Data is presented as mean ± SD (n = 3 independent spheroid cultures).

**Figure S2. Additional morphological and molecular characterization of EMOs cultured in monoculture or SPHEGaT co-culture conditions. (A)** Quantification of organoid solidity over the culture period in EMOs cultured in monoculture or SPHEGaT co-culture conditions. Lower solidity values indicate increased structural irregularity. Data are presented as mean ± S.D. from 3 independent biological replicates. Statistical analysis was performed using two-way ANOVA followed by multiple comparisons testing. *P < 0.05. **(B)** Quantification of PGR-positive nuclei expressed as a percentage of total nuclei in EMOs cultured in monoculture or SPHEGaT co-culture conditions. Each dot represents one biological replicate. Bars represent mean ± S.D. Statistical analysis was performed using an unpaired t-test. ***P < 0.001. N = 3 biological replicates. **(C)** RT-qPCR analysis of *SOX17*, *AREG*, and *GATA2* expression in EMOs cultured in monoculture or SPHEGaT co-culture conditions. Gene expression is shown as ΔCt values (Ct_target − Ct_reference, *GAPDH*) and plotted on a reversed y-axis, such that higher values correspond to higher expression. Each dot represents an independent biological replicate. Data represent n = 3 independent biological replicates. Statistical analysis was performed using two-way ANOVA followed by multiple comparisons testing. No statistically significant differences were observed

**Figure S3. Effects of steroid depletion and hormone add-back on EMO morphology and gene expression. (A)** Quantification of organoid circularity over the culture period in EMOs exposed to SPHEGaT-CM, SPHEGaT-CM_DCC, or SPHEGaT-CM_DCC+E2+P4. Lower circularity values indicate increased epithelial folding. Data are presented as mean ± S.D. (n = 3). **(B)** Quantification of organoid solidity under the same experimental conditions. Lower solidity values indicate increased structural irregularity. Data are presented as mean ± S.D. (n = 3). **(C)** RT-qPCR analysis of SOX17, AREG, and GATA2 expression in EMOs cultured under the indicated conditions. Gene expression is shown as ΔCt values normalized to GAPDH and plotted on a reversed y-axis, such that higher values correspond to higher expression. Each dot represents one biological replicate (n = 3). Statistical analysis was performed using two-way ANOVA followed by multiple comparisons testing. No statistically significant differences were observed in any of the analyzed parameters.

**Table S1. Age of postmenopausal ovarian tissue donors.** Ovarian cortical tissue used for stromal cell isolation was obtained from postmenopausal multiorgan donors. The table reports organ donor IDs and age (years) at the time of tissue procurement.

| **Organ donnor's ID** | **Age** |
| --- | --- |
| OD#33 | 58 |
| OD#36 | 56 |
| OD#44 | 50 |
| OD#54 | 56 |
| OD#59 | 49 |

**Table S2. Age of granulosa cell donors.** Granulosa cells were obtained from women undergoing assisted reproductive procedures (IVF/ICSI or fertility preservation). The table lists anonymized donor IDs, sample collection dates, birth dates, and age (years) at the time of oocyte retrieval.

| **ID number** | **Collection date** | **Birth date** | **Age** |
| --- | --- | --- | --- |
| **31881** | 20/10/25 | 17/01/91 | 34 |
| **31880** | 20/10/25 | 11/05/93 | 32 |
| **31878** | 20/10/25 | 18/03/89 | 36 |
| **31439** | 14/04/25 | 23/06/88 | 36 |
| **31437** | 14/04/25 | 10/11/84 | 40 |
| **31438** | 14/04/25 | 28/07/96 | 28 |
| **31440** | 14/04/25 | 07/05/93 | 31 |
| **31431** | 09/04/25 | 21/12/83 | 41 |
| **31428** | 09/04/25 | 11/05/88 | 36 |
| **31430** | 09/04/25 | 04/06/82 | 42 |
| **31432** | 09/04/25 | 16/03/91 | 34 |
| **31523** | 14/05/25 | 10/12/89 | 35 |
| **31522** | 14/05/25 | 20/06/86 | 38 |
| **31532** | 19/05/25 | 12/07/87 | 37 |
| **31533** | 19/05/25 | 15/03/89 | 36 |
| **31573** | 02/06/25 | 04/12/87 | 37 |
| **31571** | 02/06/25 | 18/07/83 | 41 |
| **31570** | 02/06/25 | 30/06/88 | 36 |
| **31576** | 02/06/25 | 22/07/00 | 24 |
| **31572** | 02/06/25 | 05/10/88 | 36 |

**Table S3. Age of endometrial biopsy donors.** Endometrial biopsies used for organoid generation were collected from reproductive-age women. The table lists donor IDs and age (years) at the time of biopsy.

| **Organ donnor's ID** | **Age** |
| --- | --- |
| #75 | 28 |
| #76 | 30 |
| #48 | 30 |

**Table S4. Primary and secondary antibodies used for immunofluorescence analyses.**

| **Antibody ID** | **Supplier, Cat#** | **Working Dilution** |
| --- | --- | --- |
| CYP19 Antibody (E-9) | Santa Cruz Biotechnology, TX, USA, Cat# sc-374176 | 1/200 |
| Cytochrome P450 17A1 Rabbit Polyclonal Antibody | Biorbyt, Cambridge, UK, Cat# orb5948 | 1/200 |
| Anti-CD13 antibody | Abcam, Cambridge, UK, Cat# ab7417 | 1/300 |
| Anti-FSH-R antibody | Abcam, Cambridge, UK, Cat# ab113421-1001 | 1/50 |
| Anti-Ki67 antibody | Abcam, Cambridge, UK, Cat# ab16667 | 1/200 |
| Estrogen Receptor alpha Polyclonal Antibody | Invitrogen, MA, USA, Cat# PA1-309 | 1/200 |
| Progesterone Receptor A/B (D8Q2J) XP® Rabbit mAb | Cell Signaling Technology, MA, USA, Cat# 8757T | 1/200 |
| Cytokeratin 8 Antibody (Ks8.7) | Santa Cruz Biotechnology, TX, USA, Cat# sc-101459 | 1/100 |
| CD326 (EpCAM) Monoclonal Antibody (1B7), eBioscience™ | Invitrogen, MA, USA, Cat# 14-9326-82 | 1/200 |
| Vimentin Monoclonal Antibody | Invitrogen, MA, USA, Cat# MA5-11883 | 1/300 |
| E-Cadherin Rabbit Monoclonal Antibody | Cell Signaling Technology, MA, USA, Cat# 24E10 | 1/300 |
| HIF-2 alpha/EPAS1 Antibody - BSA Free | Novus Biologicals, CO, USA, Cat# NB100-122 | 1/300 |
| Anti-Collagen IV antibody | Abcam, Cambridge, UK, Cat# ab6586 | 1/300 |
| Goat anti-Rabbit IgG (H+L) Highly Cross-Adsorbed Secondary Antibody, Alexa Fluor™ 488 | Thermo Fisher Scientific, MA, USA, Cat# A-11034 | 1/250 |
| Goat anti-Mouse IgG (H+L) Highly Cross-Adsorbed Secondary Antibody, Alexa Fluor™ 594 | Thermo Fisher Scientific, MA, USA, Cat# A-11032 | 1/250 |
| Goat anti-Rabbit IgG (H+L) Cross-Adsorbed Secondary Antibody, Alexa Fluor™ 594 | Thermo Fisher Scientific, MA, USA, Cat# A-11012 | 1/250 |

**Table S5. Primer sequences used for RT-qPCR analysis.**

| Name | Gene | Primer(forward) | Primer(reverse) |
| --- | --- | --- | --- |
| Glyceraldehyde-3-phosphate dehydrogenase | *GAPDH* | GGT ATC GTG GAA GGA CTC ATG AC | ATG CCA GTG AGC TTC CCG TTC AG |
| SRY-Box Transcription Factor 9 | *SOX9* | AGG AAG CTC GCG GAC CAG TAC | GGT GGT CCT TCT TGT GCT GCA C |
| Estrogen receptor 1 | *ESR1* | CAC TTT GAT CCA CCT GAT GGC | CTG TAC AGA TGC TCC ATG CC |
| Progesterone receptor | *PGR* | GAG TTG TGT CGA GCT CAC AGC G | GTT TCA CCA TCC CTG CCA AT |
| Secreted phosphoprotein 1 | *SPP1* | CAC ATA TGA TGG CCG AGG TG | TCC TCG CTT TCC ATG TGT GA |
| [Leukemia Inhibitory Facto](https://www.google.com/search?q=Leukemia+Inhibitory+Factor&sca_esv=e2f48ede960f4bee&sxsrf=ANbL-n5HRma4qeFh1uRUZTkQbKKHpjFxWw%3A1768810273126&ei=IedtaYG_B4XV7M8P95e4sQo&ved=2ahUKEwiUueiElJeSAxU0VKQEHcuuIs4QgK4QegQIARAD&uact=5&oq=lif+gene&gs_lp=Egxnd3Mtd2l6LXNlcnAiCGxpZiBnZW5lMgYQABgHGB4yCBAAGAcYChgeMgYQABgHGB4yBhAAGAcYHjIGEAAYBxgeMgYQABgHGB4yBhAAGAcYHjIGEAAYBxgeMgYQABgHGB4yBhAAGAcYHkjxBFAAWLUCcAB4AZABAJgBSaABwQGqAQEzuAEDyAEA-AEBmAIDoALSAZgDAJIHATOgB5sTsgcBM7gH0gHCBwMyLTPIBw2ACAA&sclient=gws-wiz-serp)r | *LIF* | AGT GCA GCC CAT AAT GAA GGT | TTG TGA CAT GGG TGG CGT AT |
| Progestagen-associated endometrial protein | *PAEP* | AGG CAC CTA TGG TAC TTG CTG | GGT TAT TCT TTG AAA GGG CAG GA |
| Glutathione peroxidase 3 | *GPX3* | CCT GAC GGG CCA GTA CAT T | CGG ACA TAC TTG AGG GTA GGA A |
| Forkhead box protein J1 | *FOXJ1* | ACT CGT ATG CCA CGC TCA TCT G | GAG ACA GGT TGT GGC GGA TTG A |
| 17β-Hydroxysteroid dehydrogenase 2 | *HSD17B2* | TCC AAC CTG GAG GCT TCC TAA C | GCT GTG CTA AGA TGT AGT CCT GG |
| Amphiregulin | *AREG* | ACT CGG CTC AGG CCA TTA TG | CCA GAA AAT GGT TCA CGC TTC |
| GATA binding protein 2 | *GATA2* | CAG CAA GGC TCG TTC CTG TTC A | ATG AGT GGT CGG TTC TGC CCA T |
| SRY-box transcription factor 17 | *SOX17* | ACG CTT TCA TGG TGT GGG CTA AC | GTC AGC GCC TTC CAC GAC TTG |
